# Development and Application of a Sexual Dysfunction Assessment Scale for Older Stroke Patients in China

**DOI:** 10.1093/geroni/igaf122.2771

**Published:** 2025-12-31

**Authors:** Shaorui Bao

**Affiliations:** The First Affiliated Hospital of Wenzhou Medical University, Wenzhou, Zhejiang, China (People’s Republic)

## Abstract

Sexual dysfunction (SD) is common among older stroke patients, affecting their physical, psychological, and social well-being. In China, SD is often overlooked due to cultural taboos around discussing sexuality in older adults. This study aimed to develop a culturally sensitive and reliable Sexual Dysfunction Assessment Scale (SDAS) for older stroke patients in China. A mixed-methods approach was used, including a literature review and expert interviews to identify factors affecting SD in this population. The SDAS was developed and tested for content validity through expert evaluation and pilot-tested on 126 older stroke patients in China. Reliability was assessed using Cronbach’s alpha (0.85), and construct validity was evaluated through factor analysis. Preliminary results showed that the SDAS effectively captured critical aspects of sexual health and was practical for use in clinical settings. This scale provides healthcare professionals with a reliable tool to identify SD early, facilitate open discussions with patients, and improve patient care. The SDAS contributes to advancing sexual health research in aging populations, with potential for use in other countries with similar cultural contexts.

